# Framing Equity and Social Justice in a New Era for Aging Centers

**DOI:** 10.1093/geroni/igaf122.613

**Published:** 2025-12-31

**Authors:** Angela Perone

**Affiliations:** University of California, Berkeley, Berkeley, California, United States

## Abstract

As our population ages and becomes more diverse, the work of aging centers is needed now more than ever before. Founded in 1996 through generous support from the Eugene and Rose Kleiner Family Foundation, the Center for the Advanced Study of Aging Services (CASAS) conducts research, educates emerging and established leaders, and informs policies to improve the lives of older adults and their care partners. As part of the School of Social Welfare at the University of California, Berkeley, CASAS is rooted in equity and social justice values. Social justice is explicitly named as a core value and ethical principle in the code of conduct for social workers through the National Association of Social Workers (NASW). Many of the contested research, education, and language stemming from prohibited work around DEI contrasts directly with professional standards for social work researchers, educators, and practitioners. This presentation will illuminate evolving challenges and strategies employed by CASAS as a case study to reconcile these inherent contradictions. Organizational auto-ethnographic data from this case study illustrates the diversity of academic, professional, and policy frames and approaches to equity and social justice in a turbulent social and political moment of uncertainty. It also provides insights for other aging centers, researchers, and practitioners navigating these professional and ethical tensions.

